# Catalytically-relevant electron transfer between two hemes *b*_L_ in the hybrid cytochrome *bc*_1_-like complex containing a fusion of *Rhodobacter sphaeroides* and *capsulatus* cytochromes *b*

**DOI:** 10.1016/j.bbabio.2013.02.007

**Published:** 2013-06

**Authors:** Monika Czapla, Ewelina Cieluch, Arkadiusz Borek, Marcin Sarewicz, Artur Osyczka

**Affiliations:** Department of Molecular Biophysics, Faculty of Biochemistry, Biophysics and Biotechnology, Jagiellonian University, 30-387 Kraków, Poland

**Keywords:** FeS subunit, subunit of cytochrome *bc*_1_ containing 2 iron-2 sulfur cluster, *R.*, *Rhodobacter*, EDTA, Ethylenediaminetetraacetic acid, HRP, horseradish peroxidase, B–B complex, cytochrome *bc*_1_-like complex containing fused cytochrome bb in the place of two separate cytochrome *b* subunits in the dimer, B_S_ complex, cytochrome *bc*_1_ complex containing cytochrome b_S_ in the place of cytochrome *b*, B_S_*–*B, cytochrome *bc*_1_-like complex containing fused cytochrome b_S_b in the place of two separate cytochrome *b* subunits in the dimer, cytochrome bb, a fusion of two cytochromes *b* of *R*. *capsulatus*, cytochrome b_S_, cytochrome *b* of *R*. *sphaeroides* expressed in *R*. *capsulatus* cells, cytochrome b_S_b, a hybrid fusion of cytochrome *b* of *R. sphaeroides* and cytochrome *b* of *R. capsulatus*, B–B strain, MT-RBC1 strain complemented with pMTS1-BBST plasmid used to express the B–B complex, B_S_ strain, MT-RBC1 strain complemented with pMTS1-BS plasmid used to express the B_S_ complex, B_S_–B strain, MT-RBC1 strain complemented with pMTS1-BSBST plasmid used to express the B_S_–B complex, Cytochrome *bc*_1_, Asymmetric mutagenesis, Fusion hybrid membrane protein, *Rhodobacter capsulatus*, *Rhodobacter sphaeroides*, Electron transfer

## Abstract

To address mechanistic questions about the functioning of dimeric cytochrome *bc*_1_ new genetic approaches have recently been developed. They were specifically designed to enable construction of asymmetrically-mutated variants suitable for functional studies. One approach exploited a fusion of two cytochromes *b* that replaced the separate subunits in the dimer. The fusion protein, built from two copies of the same cytochrome *b* of purple bacterium *Rhodobacter capsulatus*, served as a template to create a series of asymmetrically-mutated cytochrome *bc*_1_-like complexes (B–B) which, through kinetic studies, disclosed several important principles of dimer engineering. Here, we report on construction of another fusion protein complex that adds a new tool to investigate dimeric function of the enzyme through the asymmetrically mutated forms of the protein. This complex (B_S_–B) contains a hybrid protein that combines two different cytochromes *b*: one coming from *R. capsulatus* and the other — from a closely related species, *R. sphaeroides*. With this new fusion we addressed a still controversial issue of electron transfer between the two hemes *b*_L_ in the core of dimer. Kinetic data obtained with a series of B_S_–B variants provided new evidence confirming the previously reported observations that electron transfer between those two hemes occurs on a millisecond timescale, thus is a catalytically-relevant event. Both types of the fusion complexes (B–B and B_S_–B) consistently implicate that the heme-*b*_L_–*b*_L_ bridge forms an electronic connection available for inter-monomer electron transfer in cytochrome *bc*_1_.

## Highlights

► We created a hybrid fusion of cytochromes *b* from *R. capsulatus* and *R. sphaeroides.* ► Hybrid fusion replaces two cytochrome *b* subunits in a dimer of cytochrome *bc*_1_. ► Complex with hybrid fusion confirms fast electron transfer between two hemes *b*_L_.

## 1 Introduction

Cytochrome *bc*_1_ is an integral component of many biological energy conversion systems. Its role is to oxidize quinol and reduce cytochrome *c* and to couple these reactions with proton translocation across the membrane. This way it contributes to generation of protomotive force used to synthesize ATP. The enzyme is a homodimer in which each monomer consists of three, universally conserved subunits, cytochrome *c*_1_, the FeS subunit and cytochrome *b*. Each monomer embeds two catalytic quinone oxidation/reduction sites located on two opposite sides of the membrane (named the Q_o_ and Q_i_ sites) and two chains of cofactors that connect the sites together and also allow them to communicate with quinol pool in the membrane and cytochrome *c* pool outside the membrane (for recent reviews see [1–3]).

The distances between the cofactors in different monomers, as revealed by X-ray crystallography, are large enough to exclude possibility of inter-monomer electron transfer except for one point: a bridge formed by two hemes *b*_L_ which in the center of the dimer are at 14 Å edge to edge. This distance appears to be just at the limit of distances between the centers that exchange electrons within micro- to milliseconds, a timescale generally considered to be catalytically-relevant [4].

The revelation about the close distance between two hemes *b*_L_ inspired an intense discussion about possible electron transfer between the monomers and its role in a catalytic cycle. An assumption that such electron transfer exists means that the cofactor chains and catalytic sites of two monomers form an H-shaped electron transfer system that all together connects functionally all four catalytic sites of the dimer (see Fig. 5A). Indeed, the possibility of electron transfer between the hemes *b*_L_ was so appealing that it became an integral part of several models of the operation of cytochrome *bc*_1_ (see examples in refs [5–7]) and also was considered as providing potential means to diminish levels of unpaired electrons, thus lower risks of superoxide generation by cytochrome *bc*_1_ [6,8,9].

The kinetic evidence that the electron transfer between the hemes *b*_L_ takes place on a catalytically-relevant timescale came recently from the studies that used bacterial genetic systems to create asymmetrically-mutated variants suitable for functional studies. One approach was based on a fusion protein that replaced two cytochromes *b* in the dimer [10]. The fusion, built from two copies of the same cytochrome *b* of purple bacterium *Rhodobacter capsulatus*, was used to create a series of asymmetrically-mutated cytochrome *bc*_1_-like complexes (B–B). To examine the path that exclusively relies on the heme *b*_L_–*b*_L_ electron transfer, a cross-mutated variant of B–B was used in which the complementary segments of the dimer were cross-inactivated leaving the inter-monomer path as the only way connecting the catalytic sites (see scheme shown in Fig. 5B). Flash-induced electron transfer measurements performed with this mutant revealed that electron transfer between the two hemes *b*_L_ takes place on a millisecond timescale [10]. In addition, the functional connection between the catalytic Q_o_ and Q_i_ sites in this mutant was confirmed by analysis of its enzymatic activity both in the membranes and in the isolated form [11]. Another genetic approach exploited a two plasmid system with two different tags to generate and analyze the heterodimeric cross-inactivated forms of cytochrome *bc*_1_ of *R. capsulatus* [12]. This study provided independent experimental indications for the existence of the heme *b*_L_–*b*_L_ electron transfer.

The two approaches just described, together with a two-plasmid system developed for *Paraccocus denitrificans* [13], are so far the only known systems that allow studying the operation of the dimeric cytochrome *bc*_1_ through asymmetric mutagenesis. In all cases the expression of heterodimers relies on two copies of the same gene that serve as a template for mutagenesis. From the technical point of view this makes those systems challenging in that it requires a special experimental care to guard against genetic recombination to maintain the desired constructs at genetic level (see [14] for discussion on this issue). Here, we present a new system that overcomes this difficulty. The system follows our original strategy of replacing two separate cytochrome *b* subunits in the dimer with a fusion protein, however now instead of fusing two identical cytochromes *b* of *R. capsulatus* we created a hybrid protein combining two different cytochromes *b*: one coming from *R. capsulatus* and the other — from closely related *R. sphaeroides*. With this new fusion we provide further kinetic evidence for the existence of heme *b*_L_–*b*_L_ electron transfer on a catalytically-relevant timescale.

## 2 Materials and methods

### 2.1 Bacterial strains, growth conditions and plasmids

*E. coli* (HB101 and DH5α) were grown in liquid or solid Luria–Bertrani (LB) medium supplemented with appropriate antibiotics (ampicillin or kanamycin), at 37 °C. *R. capsulatus* cells were cultivated on liquid or solid mineral-peptone-yeast extract (MPYE) medium supplemented with kanamycin when needed. They were grown at 30 °C in the dark under semiaerobic conditions or in light under anaerobic conditions. Photosynthetic growth abilities were tested on MPYE plates using anaerobic jars (GasPakTM Anaerobe Container System, BD). MT-RBC1, a strain in which the chromosomal copy of *pet*ABC operon has been deleted, was used as a host for expression of cytochrome *bc*_1_ and its derivatives from expression vectors introduced into MT-RBC1 via triparental crosses [15].

The plasmids pPET1-BL [14], pUC-BLST [14], and pBC9 [16] were used as templates for genetic manipulations. The plasmid pMTS1 [15] (carries a *pet*ABC operon coding for three subunits of *R. capsulatus* cytochrome *bc*_1_) and its mutagenized derivatives were used as expression vectors.

### 2.2 Construction of expression plasmids

The plasmid pMTS1-BS used for expression of B_S_ complex (cytochrome *bc*_1_ containing *R. capsulatus* FeS and cytochrome *c*_1_ subunits and *R. sphaeroides* cytochrome *b*) was constructed from two plasmids: pPET1-BL and pBC9. The steps of pMTS1-BS construction are described in details in Supplementary data Fig. S1.

The plasmid pMTS1-BSBST used for expression of B_S_–B complex (cytochrome *bc*_1_-like complex in which two separate cytochrome *b* subunits in dimer are replaced with a hybrid cytochrome b_S_b built of *R. sphaeroides* and *R. capsulatus* cytochromes *b* fused together) and its mutagenized derivatives containing various combinations of point mutations of cytochrome *b*: G158W, H198N and H212N, were constructed according to description in Supplementary data Fig. S2.

### 2.3 Isolation of membranes and proteins, electrophoresis and Western blot

The chromatophore membranes were prepared from semiaerobically grown cultures of *R. capsulatus* as described in [17]. Membranes solubilization with n-dodecyl-β-D-maltoside (DDM) and protein purifications were performed as described in [14]. The B_S_ complex was purified using DEAE-Biogel column (BioRad), while the B_S_–B complexes were purified using Strep-tag affinity chromatography (IBA Biotagnology). Sodium dodecyl sulfate polyacrylamide gel electrophoresis (SDS-PAGE) was performed as described in [18]. The Western blot against Strep-tag was performed according to the protocol supplied by IBA with HRP-streptactin.

### 2.4 Optical and electron paramagnetic resonance spectroscopy and flash-induced electron transfer measurements

Optical spectra for *b*- and *c*-type cytochromes were recorded at room temperature using Shimadzu UV-2450 spectrophotometer. The difference spectra were obtained with samples that were first oxidized by an addition of potassium ferricyanide and then reduced by using either sodium ascorbate or a minimal amount of solid sodium dithionite. Continuous wave electron paramagnetic resonance (EPR) spectra of 2Fe-2S cluster in chromatophores were measured according to the protocol described in [19]. Flash-induced electron transfer of B_S_ and B_S_–B complexes were performed as described in [20]. For the measurements, chromatophore membranes were suspended in 50 mM MOPS buffer pH 7, containing 100 mM KCl, 1 mM EDTA, 3.5 μM valinomycin, and appropriate redox mediators (7 μM 2,3,5,6-tetramethyl-1,4-phenylenediamine, 1 μM phenazine methosulfate, 1 μM phenazine ethosulfate, 5.5 μM 1,2-naphthoquinone, 5.5 μM 2-hydroxy-1,4-naphthoquinone). The samples were poised at an ambient potential of 100 mV. Transient cytochrome *c* and *b* reduction kinetics were followed at 550–540 nm and 560–570 nm, respectively. Inhibitors antimycin A and myxothiazol were used at a final concentration of 7 μM.

## 3 Results

### 3.1 Cytochrome *b* of *R. sphaeroides* can replace native cytochrome *b* in *R.* capsulatus cytochrome *bc*_1_

**Fig. 1:**
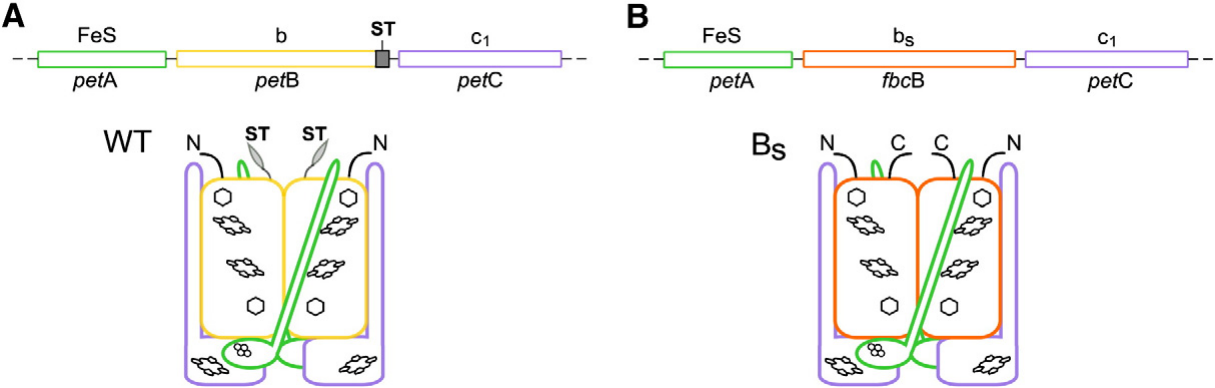
Schematic representation of operon and subunit composition of native *Rhodobacter capsulatus* cytochrome *bc*_1_ and engineered B_S_ complex. (A) Native operon *pet*ABC contains three genes coding for three catalytic subunits that assemble as a homodimer: *pet*A for FeS subunit (green), *pet*B for cytochrome *b* subunit (yellow) and *pet*C for cytochrome *c*_1_ (violet). ST − sequence coding for the Strep-tag (gray). (B) In the *pet*ABC operon, *pet*B gene was replaced by *fbc*B gene encoding cytochrome *b* subunit of *R. sphaeroides bc*_1_ complex (orange). The expression of this operon resulted in a formation of the dimeric B_S_ complex in which the cytochrome *c*_1_ and FeS subunits are from *R. capsulatus* while the cytochrome *b* subunit is from *R. sphaeroides* (orange). This cytochrome is named b_S_.

**Fig. 2:**
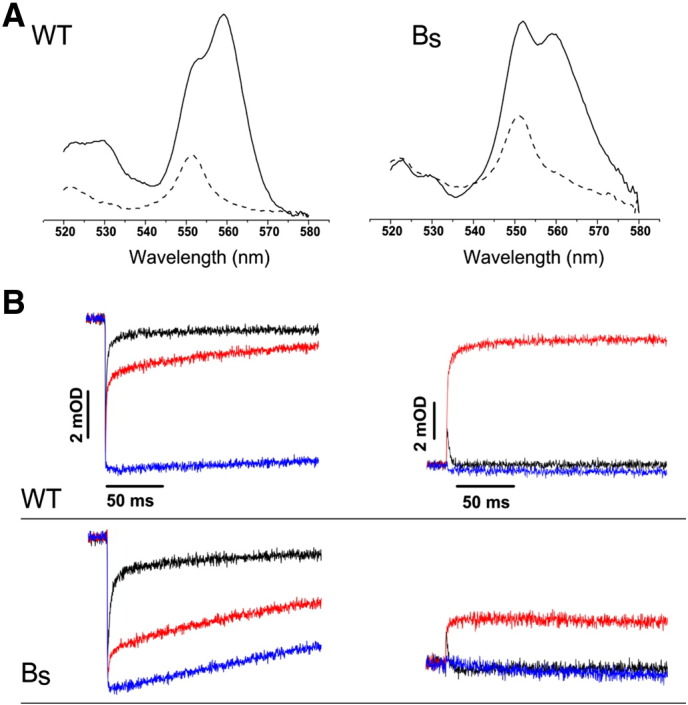
Spectroscopic and kinetic properties of B_S_ complex. (A) Reduced *minus* oxidized optical difference spectra of b- and c-type hemes in chromatophore membranes isolated from wild-type (WT) and the B_S_ strain (B_S_). Dithionite *minus* ferricyanide spectra — solid lines, ascorbate *minus* ferricyanide spectra — dashed lines. (B) Light-induced cytochrome *c* oxidation and re-reduction (left panel) and cytochrome *b* reduction and re-oxidation (right panel) of WT and B_S_ complexes recorded at 550–540 nm and 560–570 nm, respectively, at pH 7 and an ambient potential of 100 mV. Color code: no inhibitor, black; antimycin, red; and myxothiazol, blue. The vertical and horizontal scales of the lower panel are as those of the upper panel shown in the figure.

As prerequisite for experiments of fusing two different cytochromes *b* described in the following sections, we have tested the effect of replacing native cytochrome *b* of *R. capsulatus* cytochrome *bc*_1_ with that coming from the closely related strain, *R. sphaeroides*. To this end we constructed an expression vector pMTS1-BS, which in the place of native *R. capsulatus pet*B gene in *pet*ABC operon contained the gene *fbc*B coding for *R. sphaeroides* cytochrome *b* (Fig. 1). The major steps of pMTS1-BS construction are summarized in Supplementary data Fig. S1. We introduced this plasmid to *R. capsulatus* MT-RBC1 strain and found that an appropriately assembled and functional cytochrome *bc*_1_ (named B_S_) was expressed in the cells. The absorption redox difference spectra of chromatophore membranes confirmed presence of hemes b and c (peaks at 560 and 550 nm in Fig. 2A, respectively). The isolated from membranes B_S_ complexes showed similar optical spectra and contained all three catalytic subunits, cytochrome *b*, *c*_1_, and FeS, as seen on SDS-PAGE gels (not shown).

Light-induced electron transfer measurements in chromatophore membranes confirmed that B_S_ showed typical phases of electron transfer related to the action of the catalytic sites and responded in native-like manner to an addition of specific inhibitors of cytochrome *bc*_1_ (myxothiazol for the Q_o_ site and antimycin for the Q_i_ site) (Fig. 2B). From kinetic transients of cytochrome *c* reduction and cytochrome *b* re-oxidation in the absence of inhibitor it is clear that the enzyme is fully operational (both the reduction of cytochrome *c* and the re-oxidation of heme *b* proceed to completion). However the rates of the light-induced reactions were lower, comparing to the respective rates of native cytochrome *bc*_1,_ and also the amplitude of heme *b* reduction in the presence of antimycin was smaller (Fig. 2B, B_S_ vs WT). This indicates that in the presence of this inhibitor the distribution of electrons within the cofactor chains is altered in B_S_ (see ref. [20]). The reason of these effects is currently unknown but may reflect some structural distortions associated with a necessity to accommodate *R. sphaeroides* cytochrome *b*. For example, the lack of subunit *IV*, which in native cytochrome *bc*_1_ of *R. sphaeroides* naturally interacts with cytochrome *b* subunit [21] but is missing in native cytochrome *bc*_1_ of *R. capsulatus*, and thus is not present in B_S_ either, may contribute to these effects. (Detailed analysis of B_S_ is under way and will be a subject of separate studies.) Consistent with the kinetic results, the strain expressing B_S_ showed Ps + phenotype indicating that this enzyme is functional in vivo.

Based on these results we concluded that cytochrome *b* of *R. sphaeroides* is capable of replacing native cytochrome *b* in *R. capsulatus* cytochrome *bc*_1_, and the engineered B_S_ retains functional and structural properties of cytochrome *bc*_1_.

### 3.2 Hybrid cytochrome b_S_b obtained by fusion of cytochromes *b* from *R. sphaeroides* and *R. capsulatus* assembles with other core subunits to form B_S_–B complex

**Fig. 3:**
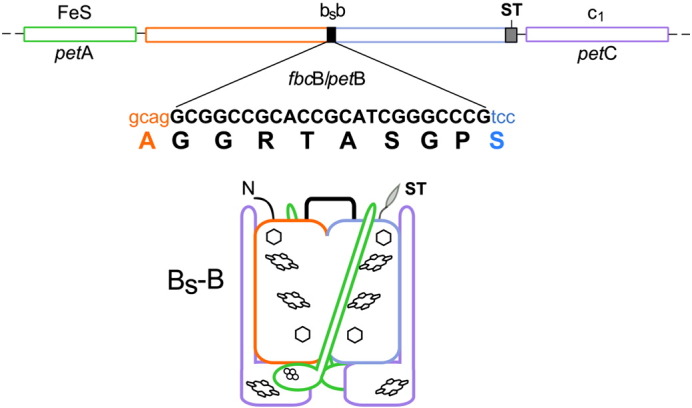
Operon organization and subunit composition of the fusion hybrid B_S_–B complex. In the *pet*ABC operon, the *pet*B gene was replaced by a hybrid fusion gene *fbcB/petB* composed of *fbc*B of *R. sphaeroides* and *pet*B of *R. capsulatus* (orange and blue). The remaining genes in the operon are: *pet*A coding for the FeS subunit (green), and *pet*C coding for cytochrome *c*_1_ (violet). The expression of this operon resulted in a formation of the B_S_–B complex in which the two cytochrome *b* subunits in the dimer are replaced with hybrid cytochrome b_S_b (a fusion of cytochromes *b* of *R. sphaeroides* and *R. capsulatus*). DNA sequence and amino acid composition of the linker is shown in black. Orange and blue letters indicate the last and the first codons/amino acid residues that were left unchanged in *R. sphaeroides* and *R.capsulatus* gene/protein, respectively. ST − sequence coding for the Strep-tag (gray).

The fusion of cytochromes *b* from *R. capsulatus* and *R. sphaeroides* was achieved adopting similar strategy that was previously used to fuse two cytochromes *b* of *R. capsulatus* [10]. At genetic level, this strategy requires modification of the expression vector so that the operon coding for cytochrome *bc*_1_ contains cytochrome *b* gene (*pet*B) extended in frame with an additional copy of cytochrome *b* gene. While in our previous work, the two halves of the fusion gene were alike, each containing a sequence of the same *pet*B gene originated from *R. capsulatus* [10], in this work we constructed a fusion gene assembled from two different genes: *fbc*B and *pet*B (Fig. 3). The first half of the fusion gene contained the sequence of *fbc*B from *R. sphaeroides* while the second half contained the sequence of *pet*B from *R. capsulatus* with the sequence encoding Strep-tag at its 3′ end (this fusion gene was named *fbc*B*/pet*B). The other two genes of the operon *pet*ABC, encoding the FeS subunit and cytochrome *c*_1_, were left unchanged. All three genes were expressed using a vector pMTS1-BSBST, which was a derivative of pMTS1 containing *fbc*B*/pet*B in the place of *pet*B gene. The major steps of construction of pMTS1-BSBST are described in Supplementary data Fig. S2.

**Fig. 4:**
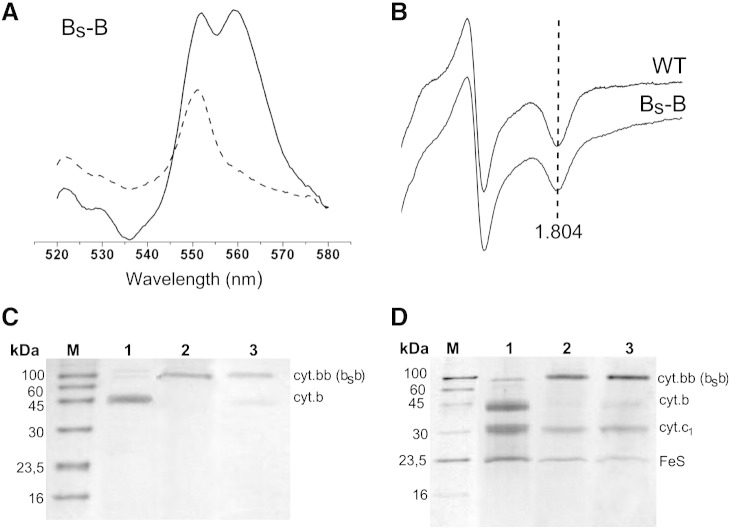
Spectroscopic properties and Western blot/SDS-PAGE analyses of B_S_–B. (A) Optical redox difference spectra of b- and c-type hemes in chromatophore membranes isolated from the B_S_–B strain. Dithionite *minus* ferricyanide spectra — solid lines, ascorbate *minus* ferricyanide spectra — dashed lines. (B) X-band continuous wave EPR spectra of the 2Fe-2S cluster of WT and B_S_–B complexes in chromatophore membranes. Dotted line shows the position of g_x_ transition. (C) Western blot against Strep-tag II (IBA Biotagnology) of chromatophore membranes isolated from wild type (lane 1), the B–B strain (lane 2) and the B_S_–B strain (lane 3). M, Molecular weight marker. Names cyt. bb and cyt. b_S_b depict the protein shown in line 2 and 3, respectively. (D) Coomassie blue stained SDS-PAGE analysis of complexes isolated using affinity chromatography (Strep-tag) from wild-type (lane 1), the B–B strain (lane 2) and the B_S_–B strain (lane 3). M, Molecular weight marker. Names cyt. bb and cyt. b_S_b are the same as in C.

Fig. 4 summarizes the results of expression of pMTS1-BSBST in MT-RBC1 cells. First, spectroscopic measurements of membranous fractions revealed the presence of redox cofactors characteristic for cytochrome *bc*_1_-type complexes: absorption redox difference spectra showed presence of hemes b and c (peaks at 560 and 550 nm in Fig. 4A, respectively) while EPR showed presence of Rieske protein appropriately interacting with occupants of the Q_o_ site [22] (characteristic g_x_ value of the spectrum of Fig. 4B). Second, Western blots revealed the presence of the fusion protein of correct size (two times larger than cytochrome *b*) in the membranes (Fig. 4C). The fusion protein was also clearly visible on SDS-PAGE of complexes isolated from the membranes using affinity chromatography (Fig. 4D). The electrophoretic profile of isolated complexes showed that the fusion protein was accompanied by the two remaining subunits of cytochrome *bc*_1_: cytochrome *c*_1_ and the FeS subunit, consistent with spectroscopic features just described. These results provided first indication that the membranes contained a cytochrome *bc*_1_-like complex built of the hybrid fusion protein (named cytochrome b_S_b) assembled together with cytochrome *c*_1_ and the FeS subunit (the entire complex was named B_S_–B). Further kinetic experiments confirmed that B_S_–B did assemble in the membranes (see below). In the remaining text the system of expression of B_S_–B in *R. capsulatus* cells will be referred as the *sphaer–caps* system. For consistency, the system of expression of B–B described earlier in [10,14] will be named as the *caps–caps* system.

We note that Western blot and Coomassie blue-stained gels revealed also traces of a protein in size corresponding to native cytochrome *b* (Fig. 4C, D). The amount of this cytochrome *b* in relation to the fusion protein was always significantly smaller, as exemplified on gel in Fig. 4D. These results indicate that in addition to the dominant fraction of B_S_–B, the membranes contain a small fraction of cytochrome *b* either alone or assembled with cytochrome *c*_1_ and FeS subunits. At present, the origin of this phenomenon is not clear. Given that the fusion constructs are commonly reported to encounter problems with proteolysis upon expression and/or isolation of proteins [23–27], we favor an explanation that in our case it is also a result of partial degradation of protein, especially of a foreign *R. sphaeroides* portion of the fusion protein with retention of the *R. capsulatus* part (this part contains Strep-tag used for Western blot detection and affinity chromatography). At the same time we are certain that this cannot be due to genetic recombination leading to shortening of the fused gene. The results shown in the next paragraphs have demonstrated that the *sphaer–caps* system exhibits high genetic stability.

Our initial attempts to eliminate completely this background of cytochrome *b* have proven that this was not a straightforward task. We did not investigate this issue any further as for the main purpose of this work it was not necessary. We reasoned that as long as B_S_–B complex was assembled in the membranes and there was a possibility to perform all appropriate control experiments, the background of cytochrome *b* would not compromise the kinetic experiments that were a subject of present studies.

### 3.3 B_S_–B protein accommodates several point mutations introduced in symmetric and asymmetric patterns

**Fig. 5:**
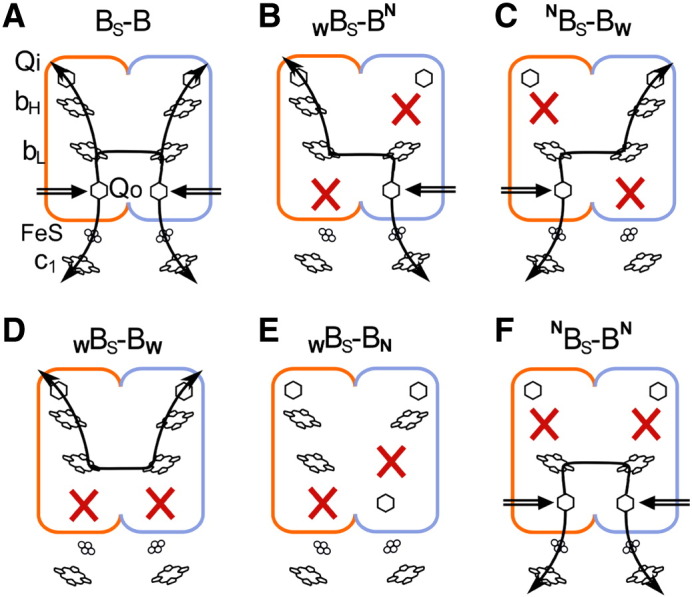
Symmetric and asymmetric knockout patterns in the fusion hybrid B_S_–B complex. (A) B_S_–B complex without mutation — four branches for electron transfer are open forming an H-shaped electron transfer system characteristic for the intact wild-type cytochrome *bc*_1_; (B) _W_B_S_–B^N^ and (C) ^N^B_S_–B_W_ — two branches across removed (cross-inactivation) and heme *b*_L_–*b*_L_ connection maintained; (D) _W_B_S_–B_W_ — both lower branches removed; (E) _W_B_S_–B_N_ — two branches across removed and heme *b*_L_–*b*_L_ connection disrupted; (F) ^N^B_S_–B^N^ — both upper branches removed (note that this form is drawn schematically but was not obtained as a fusion protein complex). W, N (subscript), and N (superscript) refer to G158W, H198N, and H212N point mutations in cytochrome *b*, respectively. Black arrows indicate functional branches. Black double arrows indicate electron entry point at the Q_o_ site. Red crosses display distribution of G158W, H198N, and H212N point mutations in B_S_–B complexes.

**Fig. 6:**
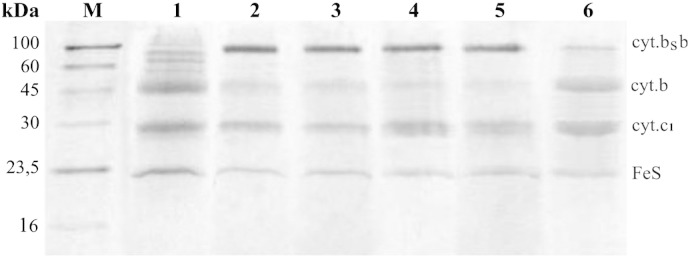
SDS-PAGE analysis of various B_S_–B complexes with knockout mutations isolated using affinity chromatography (Strep-tag). Lanes: M, Molecular weight marker (IBA); 1, wild-type cytochrome *bc*_1_; 2, _W_B_S_–B^N^; 3, ^N^B_S_–B_W_; 4, _W_B_S_–B_W_; 5, _W_B_S_–B_N_; 6, ^N^B_S_–B^N^.

In the next series of experiments, we introduced point mutations to pMTS1–BSBST template repeating the strategy used earlier to create symmetrically and asymmetrically mutated B–B complexes [10] (Fig. 5). The point mutations included G158W to inactivate the Q_o_ site and the lower branch of the H-shaped electron transfer system [22] and H212N to inactivate the Q_i_ site and the upper branch of this system [8] (numbering corresponds to the sequence of *R. capsulatus* cytochrome *b*). The asymmetric combinations contained an equivalent of G158W in one half of the hybrid gene and an equivalent of H212N in the other half (to obtain _W_B_S_–B^N^ or ^N^B_S_–B_W,_ Fig. 5B, C). The symmetric combinations contained equivalents of G158W or H212N in both halves of the gene (to obtain _W_B_S_–B_W_ or ^N^B_S_–B^N^, Fig. 5D, F). Table 1 and Fig. 6 summarize the results of expression of pMTS1–BSBST derivatives containing appropriate mutations in MT-RBC1 strain. Table 1 also compares these results with those obtained previously when the same combinations of mutations were tested with the *caps–caps* system [10,14].

From Table 1 and Fig. 6, it is clear that B_S_–B complex containing the fusion protein is assembled in all cases, except for ^N^B_S_–B^N^. Most importantly, both asymmetric combinations _W_B_S_–B^N^ and ^N^B_S_–B_W_ resulted in an assembly of the fusion protein. The *sphaer–caps* system allowed also for an assembly of the complex containing one of the symmetric mutation patterns (i.e. _W_B_S_–B_W_). The latter pattern was previously unavailable with the *caps–caps* system, which in general did not tolerate the presence of the same mutation in both halves of the fusion protein [14].

In this work we also tested a new asymmetric pattern _W_B_S_–B_N_ or _W_B–B_N_ (for *sphaer–caps* or *caps–caps* system, respectively) (Fig. 5E). This combination contained G158W in the first half of the fusion protein and an equivalent of H198N introduced in the second half and was specifically designed to perform a series of genetic and kinetic control experiments described in next paragraphs. In cytochrome *b*, mutation H198N replaces one of the histidine ligand to iron of heme *b*_L_ with non-competent asparagine and, as previous studies with *R. sphaeroides* have indicated, results in an assembly of the cytochrome *bc*_1_ complex with an impaired Q_o_ site [28]. This mutation has not been described earlier for *R. capsulatus*, but our initial experiments confirmed that also in this species H198N mutant assembles as cytochrome *bc*_1_ with impaired Q_o_ site (manuscript in preparation). We thus used H198N and G158W to create a form intended to disable both the Q_o_ sites, each by a different point mutation. Those two point mutations were separated from each other in DNA sequence of *pet*B, which was important from a genetic point of view for the planned experiments (this separation was one of the reasons for selection of H198N over other mutations in cytochrome *b* that are also known to inactivate the Q_o_ site but are closer to G158W in sequence).

As Table 1 indicates, the form containing equivalents of H198N and G158W assembled as fusion protein only as _W_B_S_–B_N_ in the *sphaer–caps* system. For the *caps–caps* system, the results were similar to those previously described for all symmetrically mutated forms (^N^B–B^N^, _W_B–B_W_) [14].

In general, from the comparison shown in Table 1 it appears that B_S_–B have more structural flexibility to accommodate larger number of mutational patterns than B–B. Possibility to analyze those combinations of B_S_–B that were previously unavailable with B–B (i.e. _W_B_S_–B_W_, _W_B_S_–B_N_) is highly valuable as it offers additional level of control and means to verify conclusions drawn earlier with B–B.

### 3.4 The *sphaer–caps* system exhibits high genetic stability

**Fig. 7:**
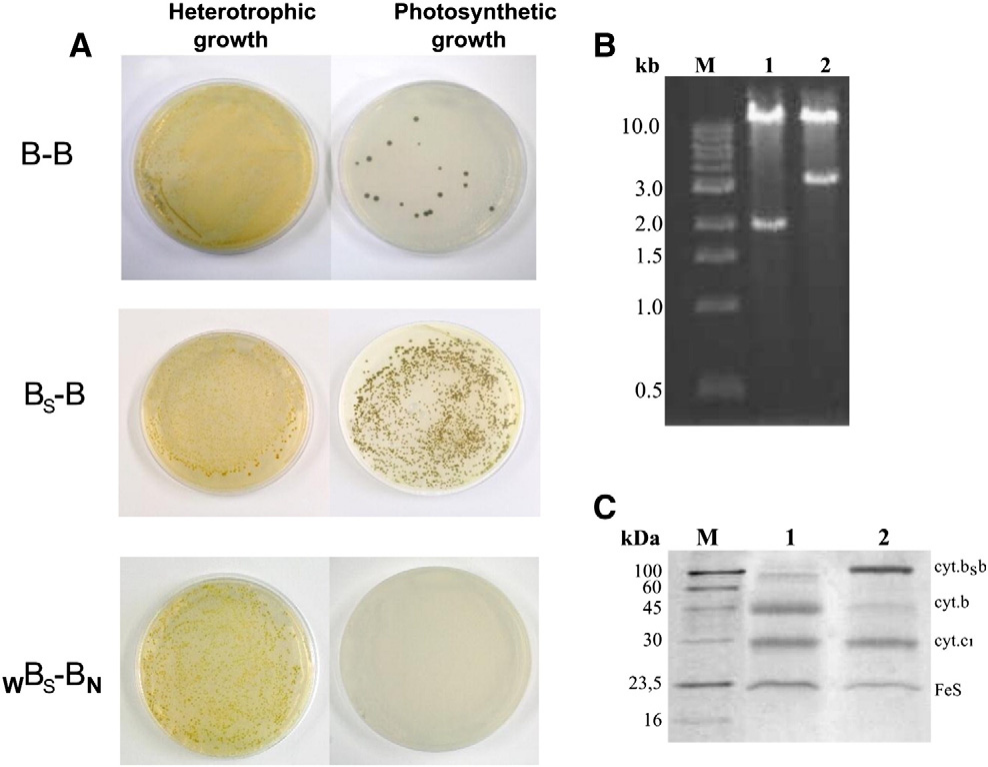
Effect of photosynthetic selection on strains expressing various fusion proteins. (A) Comparison of heterotrophic and photosynthetic growth of *R. capsulatus* strains expressing B–B (top), B_S_–B (middle), _W_B_S_–B_N_ (bottom). For each strain the same amount of cells was plated for heterotrophic and photosynthetic growth. (B) Restriction analysis of expression plasmids isolated from the B–B and B_S_–B strains grown under photosynthetic anaerobic condition (lanes 1 and 2, respectively). The presence of ~ 3.3 kb DNA fragment indicates that the plasmid bears fusion gene *fbc*B/*pet*B, while ~ 2 kb DNA fragment corresponds to the native form of gene *pet*B. M, Molecular weight marker. (C) Coomassie blue stained SDS-PAGE analysis of complexes isolated from the B_S_–B strain grown under photosynthetic conditions (lane 2) in comparison with wild-type cytochrome *bc*_1_ (lane 1). M, Molecular weight marker.

We have previously observed that in the *caps–caps* system, the cells carrying genes coding for B–B did not grow photosynthetically (exhibited Ps − phenotype). However, the photosynthetic growth conditions allowed for selection of revertants. The cells that regained Ps + phenotype carried plasmids containing only a short version of the gene (corresponding in size to a single copy of *pet*B) (Fig. 7B). The reversions to Ps + occurred with a frequency of 10^− 3^–10^− 4^, which was estimated from the number of cells that were able to grow photosynthetically at given concentration of cells. The tests involved serial dilutions experiments where the number of colonies that can grow under photosynthetic conditions was compared to the total number of cells equal to the number of cells growing under aerobic conditions (an example of the result for a given concentration of cells is shown in Fig. 7A, top).

Similar tests were now performed for the cells carrying pMTS1–BSBST (used for expression of B_S_–B) in the *sphaer–caps* system. First, we checked the cells expressing B_S_–B without additional mutations and observed that the number of colonies that grow under photosynthetic and aerobic conditions for given concentrations of the cells was always similar (Fig. 7A, middle). We also found that the cells grown under photosynthetic conditions retained the original plasmid pMTS1–BSBST with intact fused gene *fbc*B*/pet*B and showed no signs of a short copy of the gene (Fig. 7B). Furthermore, the SDS profile of complexes isolated from the membranes of these cells (Fig. 7C, lane 2) indicated that they contained B_S_–B complex with fusion protein (the SDS profile of the complexes obtained from the photosynthetic cultures was very similar to that obtained from the semiaerobic cultures, see, lane 3 of Fig. 4D). This all was a first indication that the frequency of genetic recombination in the *sphaer–caps* system is low and that this system is genetically more stable than the *caps–caps* system.

We note that because the cells expressing B_S_–B show some background of unfused cytochrome *b* subunit (Fig. 4C, D), the Ps + phenotype in itself cannot be used as an argument in discussions about possible functionality of B_S_–B in vivo. Clearly, other experiments are needed to asses it (such experiments are currently under way).

To further asses genetic stability of the *sphaer–caps* system we analyzed the non-functional _W_B_S_–B_N_ variant which had two of its Q_o_ sites disabled by two different mutations positioned in protein sequence 40 amino acids apart (G158W in one half and an equivalent of H198N in the other). In this case, although the mutant cells expressing _W_B_S_–B_N_ complex were Ps −, as expected, there was a theoretical possibility of homologous recombination between parts of the fusion gene resulting in a sequence that would remove deleterious mutation and restore the functional Q_o_ site. Those types of recombinant cells can be selected by growing the cells under photosynthetic conditions. Remarkably, however, we could not obtain recombinant cells for this mutant even when very high concentrations of cells were tested. As exemplified in Fig. 7A, bottom, all the cells exhibited Ps − phenotype. Based on the serial dilution tests [14] it was estimated that the frequency of recombination is below 10^− 6^. These results provide evidence that the *sphaer–caps* system exhibits high genetic stability. The frequency of reversion in this system is clearly orders of magnitude lower from that estimated for the *caps–caps* system [14]. We note that it also appears to be lower from the frequency of reversions estimated for the alternative two-plasmid system [12].

### 3.5 Light-induced electron transfer in B_S_–B derivatives confirm occurrence of fast electron transfer between two hemes b_L_

**Fig. 8:**
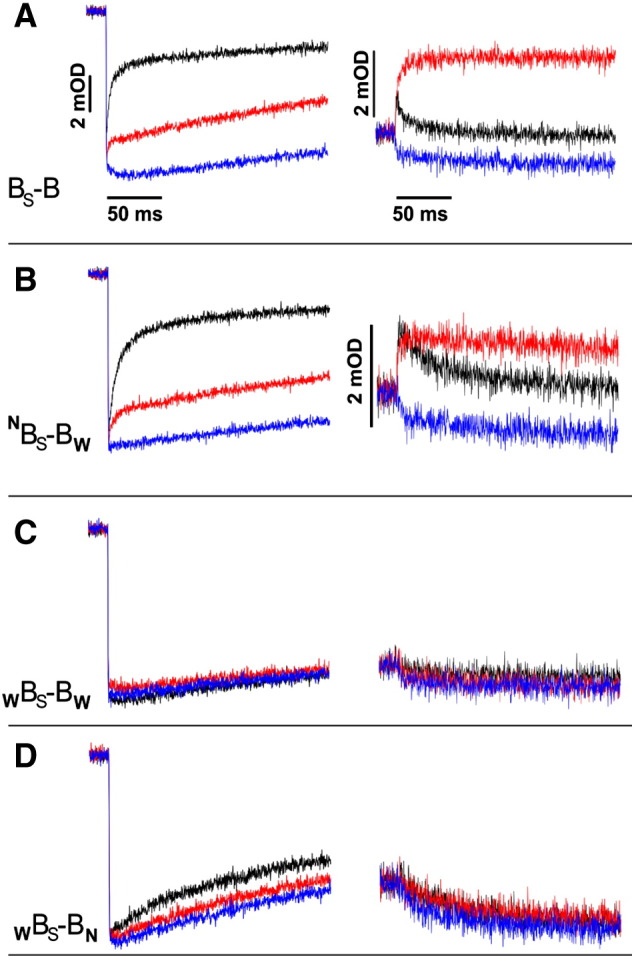
Light-induced cytochrome *c* oxidation and re-reduction (left panel) and cytochrome *b* reduction and re-oxidation (right panel) in chromatophore membranes containing B_S_–B (A), cross-inactivated ^N^B_S_–B_W_ (B) and two controls: _W_B_S_–B_W_ and _W_B_S_–B_N_ (C and D, respectively). The traces were recorded at 550–540 nm and 560–570 nm for cytochrome *c* and *b*, respectively, at pH 7 and an ambient potential of 100 mV. Color code: no inhibitor, black; antimycin, red; and myxothiazol, blue. In each panel, the vertical and horizontal scales for cytochrome *c* are as shown in A. The horizontal scales for all cytochrome *b* are as shown in A. The vertical scale for cytochrome *b* in C and D is as in B.

Fig. 8 compares kinetic traces of light-induced electron transfer in chrompatophore membranes containing various forms of B_S_–B. In the chromatophores containing B_S_–B complex without any additional mutations in the absence of any inhibitors, hemes *c* (*c*_1_ and *c*_2_) of cytochromes *c* were rapidly photo-oxidized then reduced, while heme *b*_H_ of cytochrome *b* was rapidly reduced and re-oxidized (Fig. 8A). Antimycin, inhibitor of the Q_i_ site, greatly diminished heme *c* reduction phase and fully abolished heme *b* re-oxidation phase leaving only its reduction phase. Myxothiazol, inhibitor of the Q_o_ site, abolished heme *c* reduction phase and also fully abolished heme *b* reduction and re-oxidation phases. From this data it is clear that B_S_–B exhibits all phases of electron transfer reminiscent of the functional catalytic Q_o_ and Q_i_ sites connected together, as known for the wild-type enzyme and described earlier for B–B [10].

Most significantly, asymmetric ^N^B_S_–B_W_ exhibited kinetic behavior consistent with the same mode of operation (Fig. 8B). Again, there was a clear and large phase of antimycin-sensitive reduction of hemes *c*. Heme *b* reduction and re-oxidation phases were antimycin-sensitive: in the absence of any inhibitors heme *b*_H_ was rapidly reduced and re-oxidized, while in the presence of antimycin re-oxidation phase was eliminated and only reduction phase was observed. Similar kinetic results were obtained for the mirror asymmetric form _W_B_S_–B^N^ (not shown).

In the asymmetric form ^N^B_S_–B_W_ the functional connection between the Q_o_ and Q_i_ sites can only be accomplished if electrons are transferred between the hemes *b*_L_. This is because in this mutant electrons enter the b chain (reflected as flash-induced reduction of heme *b*_H_) only through one active Q_o_ site and leave this chain (reflected as flash-induced oxidation of heme *b*_H_) only through one active Q_i_ site, but each of these two sites is located on a separate half of the fusion protein (Fig. 5C). Thus, to reach the active Q_i_ site, electrons that entered the enzyme through the active Q_o_ site must use the path: heme *b*_L_–heme *b*_L_–heme *b*_H_. This also means that when the Q_i_ site is inactive (in the presence of antimycin), heme *b*_H_ cannot be reduced in flash experiments unless the quinol-derived electron is transferred from one heme *b*_L_ (that associated with active Q_o_ site) to another heme *b*_L_ (that associated with inactive Q_o_ site).

A profound antimycin-sensitive phase of cytochrome *c* reduction and the Q_i_-site-mediated re-oxidation of heme *b*_H_ seen in ^N^B_S_–B_W_ indicate that the functional connection between the Q_o_ and Q_i_ sites is preserved in this mutant. At the same time, the reduction of heme *b*_H_ in the presence of antimycin confirms that this heme is reducible by electrons coming from the active Q_o_ site. We note that consistent with hemes *c* and *b* reduction/oxidation kinetics, ^N^B_S_–B_W_ displayed all cytochrome *bc*_1_-related phases of carotenoid band shifts typical for native cytochrome *bc*_1_ confirming full turnover of the cross-inactivated enzyme in the absence of any inhibitors (not shown). Thus, in light of the above considerations, kinetic traces of the asymmetric ^N^B_S_–B_W_ clearly indicate that electron transfer between two hemes *b*_L_ must take place on catalytically-relevant timescale. This result is fully consistent with our earlier demonstration of existence of heme *b*_L_–*b*_L_ electron transfer reported for cross-inactivated _W_B–B^N^ constructed using the *caps–caps* fusion system [10].

We note that re-reduction of cytochromes *c* in the absence of inhibitors reaches similar level in both ^N^B_S_–B_W_ and B_S_–B, but at the same time the amplitude of heme *b*_H_ reduction in ^N^B_S_–B_W_ in the presence of antimycin is smaller comparing to the respective amplitude of B_S_–B (Fig. 8B and A). This indicates that, in the post-flash redistribution, electrons equilibrate on cofactors chains to the same final levels in ^N^B_S_–B_W_ and B_S_–B as long as the Q_o_ and Q_i_ sites communicate with the quinone and cytochrome *c* pools. On the other hand, when the outflow of electrons through the Q_i_ site is blocked by antimycin the final distribution of electrons is different and reduction of heme *b*_H_ in ^N^B_S_–B_W_ is less complete. At this stage deciding what causes this shift in the final equilibrium is difficult, however this result should not be considered unexpected, especially in light of similar changes in electron distribution observed in antimycin-inhibited cytochrome *bc*_1_ when a barrier for a particular electron transfer reaction was specifically modified [20].

Fig. 8C, D show the results of the controls that involved two fusion forms designed to have heme *b*_L_–*b*_L_ electron transfer eliminated: _W_B_S_–B_W_ and _W_B_S_–B_N_ (Fig. 5D and E, respectively). These controls deserve particular attention as, for reasons discussed earlier in [14], they were not previously available with the *caps–caps* fusion system.

In _W_B_S_–B_W_, no kinetic phases of heme *c* reduction or heme *b* reduction and re-oxidations were observed (Fig. 8C). Furthermore, addition of either antimycin or myxhotiazol had no effect on the kinetic traces. These results are similar to the effects of G158W [10,22] and report that both the Q_o_ sites in _W_B_S_–B_W_ are inactive. _W_B_S_–B_W_ demonstrates that the complex containing the fusion protein can be fully inactivated when the Q_o_-site-inactivating mutation is present in both of its halves.

Kinetic traces recorded for the second control, _W_B_S_–B_N,_ are shown in Fig. 8D. The heme *c* reduction phase was almost fully suppressed (we note that the residual cytochrome *c* reduction kinetics seen in the absence of any inhibitors must have come from the half of _W_B_S_–B_N_ containing the equivalent of H198N, as we also observed such residual activity in the H198N mutant) and there were no signs of any phases of heme *b* reduction or re-oxidation. Furthermore, the traces recorded for heme *b*_H_ were not sensitive to antimycin or myxhotiazol. Clearly, _W_B_S_–B_N_ shows no signs of functional connection between the Q_o_ and Q_i_ sites nor electron transfer between two hemes *b*_L_. We emphasize that the kinetic traces of _W_B_S_–B_N_ are clearly different from those of ^N^B_S_–B_W_ (Fig. 8D vs B).

The form _W_B_S_–B_N_, adds to _W_B_S_–B_W_ as another version of control with both of the Q_o_ sites inactivated (Fig. 5D, E). But in the case of _W_B_S_–B_N_, unlike in _W_B_S_–B_W,_ there exists a possibility of genetic recombination between parts of the fusion gene to obtain pseudo-native form of the enzyme. Occurrence of such reversions at significant level would manifest itself as a background of native-like kinetic traces visible in flash-induced electron transfer measurements. The results obtained with _W_B_S_–B_N_ clearly demonstrate that this is not the case. The lack of any background of native-like kinetics in chromatophores containing _W_B_S_–B_N_ is consistent with the observation that the *sphaer–caps* system exhibits high genetic stability, thus recombinations between parts of the fused gene that would obscure the kinetic results do not occur.

Fig. 5F presents schematically the cofactor pattern in the mutant allowing to test the conditions when the active Q_o_ site mediates reduction of heme *b*_L_ but further electron transfer to heme *b*_H_ is prevented. Although such a mutant was not obtained as fusion protein complex (see Fig. 6, lane 6), the characteristic light-induced kinetic transients of the corresponding b_H_ knockout (cytochrome *bc*_1_ mutant lacking both hemes *b*_H_) [8] are available for comparison with the transients of ^N^B_S_–B_W_. As described previously [10], and also shown in Fig. S3A, the b_H_ knockout does not exhibit profound antimycin-sensitive phase of cytochrome *c* reduction present in wild-type cytochrome *bc*_1_ (Fig. 2B) and in ^N^B_S_–B_W_ (Fig. 8B). The b_H_ knockout neither shows heme *b*_H_ reduction/re-oxidation phases in the absence of inhibitors, nor heme *b*_H_ reduction in the presence of antimycin (Fig. S3B), as observed at 560–570 nm in wild-type cytochrome *bc*_1_ (Fig. 2B) and in ^N^B_S_–B_W_ (Fig. 8B). The involvement of heme *b*_L_ in the *b*_H_ knockout can be seen as antimycin-insensitive reduction phase at 566–573 nm (Fig. S3C). The clear differences between the kinetic traces of the *b*_H_ knockout with that of ^N^B_S_–B_W_ rule out the possibility that the latter ones result from electron transfer reactions involving just heme *b*_L_ without participation of heme *b*_H_ (and the Q_i_ site in the absence of inhibitors).

To sum up the results of control experiments, the kinetic traces recorded for two control fusion forms _W_B_S_–B_W_ and _W_B_S_–B_N_ and for the *b*_H_ knockout show no signs of functional connection between the catalytic Q_o_ and Q_i_ sites, nor the heme *b*_H_ reduction/reoxidation reminiscent of heme *b*_L_–*b*_L_ electron transfer (Fig. 8C, D). This further substantiates the conclusion that the kinetic traces of the cross-inactivated asymmetric form ^N^B_S_–B_W_ (Fig. 8B) do reveal heme *b*_L_–*b*_L_ electron transfer and functional connection between the catalytic sites.

## 4 Discussion

Our earlier work has shown that a genetic approach of fusing two cytochrome *b* subunits in cytochrome *bc*_1_ offers an attractive opportunity to address crucial bioenergetic questions related to the mechanisms of operation of this enzyme. This in particular concerned controversial issues of possible allostery within the dimeric complex and possibility of communication between the monomers. Experimental results addressing these points have demonstrated that monomers operate independently, but at the same time — can exchange electrons using the electron-transfer bridge formed by two hemes *b*_L_ in the core of the dimer [10,11,14].

Mechanistic conclusions were drawn from kinetic analysis of the mutants containing a fusion protein (cytochrome bb) assembled with other core subunits to form cytochrome *bc*_1_-like complex named B–B. The point mutations introduced to cytochrome bb enabled inactivation of individual segments of cofactor chains in various symmetric and asymmetric combinations, exposing various electron transfer paths within B–B for kinetic testing. The path that specifically exposed the electron transfer between two hemes *b*_L_ was identified in the asymmetric form _W_B–B^N^, in which the complementary parts of the fusion protein were cross-inactivated [10].

From the protein engineering point of view this fusion system (referred in this paper as the *caps–caps* system) came as a remarkable example of flexibility within the whole protein expression and assembly system, which clearly was able to adopt itself to accommodate B–B and several of its mutant derivatives. Because, however, the fusion was based on the two copies of the same gene, the risk of genetic recombination (to remove one copy of a gene or exchange complementary fragments of a gene) imposed a necessity of experimental care to implement protocols that ensured that samples used for kinetic analysis were devoid of unwanted background of recombined proteins. This present work provides an attractive alternative template for asymmetric mutagenesis: a cytochrome *bc*_1_-like complex with a new fusion protein expressed from a gene of improved genetic stability. This new system allowed us to obtain a whole family of mutants that included the cross-inactivated variants together with an extended set of control forms to further analyze electronic communication between two hemes *b*_L_.

The system was based on a fusion of two cytochromes *b*, one coming from *R. sphaeroides* and the other from *R. capsulatus* (the *sphaer-caps* system). Because the new fusion comprised two different genes, the *sphaer–caps* system turned out to be genetically more stable than the *caps–caps* system. Indeed, 17.6% of difference between the two genes in the *sphaer–caps* system appeared sufficient to lower the frequency of recombination between the genes [29,30] orders of magnitude in comparison to the *caps–caps* system. At the same time, the structure of those two closely related cytochromes is very similar [31,32] (90.4% of similarity based on primary sequence) and, as we have shown here, not only cytochrome *b* of *R. sphaeroides* can replace native cytochrome *b* in *R. capsulatus* cytochrome *bc*_1_, but also hybrid cytochrome b_S_b (a fusion of *R. sphaeroides* and *R. capsulatus* cytochromes *b*) assembled with other subunits in membranes of *R. capsulatus* cells to form a hybrid cytochrome *bc*_1_-like complex. This latter complex, named B_S_–B, corresponds to the previously described B–B [10].

Using B_S_–B as a template we prepared the cross-inactivated variants ^N^B_S_–B_W_ and _W_B_S_–B^N^ which repeated the asymmetric combination of mutations in _W_B–B^N^ originally used to test the electron transfer between the two hemes *b*_L_. In addition, we prepared the control forms _W_B_S_–B_W_ and _W_B_S_–B_N_, which had both of the Q_o_ sites of the complex inactivated by mutations and thus allowed us to test the conditions when electron transfer between two hemes *b*_L_ was not possible within the fusion protein.

The flash-induced experiments performed with ^N^B_S_–B_W_ and _W_B_S_–B^N^ showed the presence of kinetic phases reminiscent of the functional connection between the Q_o_ and Q_i_ sites. In addition, these experiments revealed reduction of heme *b*_H_ in the presence of antimycin. As all these reactions in those mutants can only be accomplished if the heme *b*_L_–*b*_L_ electron transfer takes place, it is clear that the kinetic traces proved that these two hemes exchange electrons on a catalytically-relevant timescale. ^N^B_S_–B_W_ and _W_B_S_–B^N^ demonstrated this reaction in the same manner as the previously described _W_B–B^N^ [10].

On the other hand, the traces of _W_B_S_–B_W_ and _W_B_S_–B_N_ showed neither signs of functional connection between the Q_o_ and Q_i_ sites nor signs of reduction of heme *b*_H_ in the presence of antimycin, confirming that the heme *b*_L_–*b*_L_ electron transfer does not occur in those two mutants. The observation that the kinetic traces of those two controls clearly differ from the traces of cross-inactivated ^N^B_S_–B_W_ and _W_B_S_–B^N^ further substantiated the conclusion that the latter ones did reveal heme *b*_L_–*b*_L_ electron transfer.

The lack of electron transfer between two hemes *b*_L_ in kinetic traces of _W_B_S_–B_N_ deserves particular emphasis, as this is a variant in which the recombination between the parts of fusion gene to restore the functional Q_o_ site was theoretically possible. Such recombination was described recently by Hong et al. [33] who constructed similar types of mutants _N_B–B^N^ or ^N^B–B_N_ in *R. sphaeroides* cells (in those forms the complex was fully inactivated by mutations H198N and H212N) and observed that they grew photosynthetically. As the photosynthetic growth in those cases must have come from the reverted forms that were effectively selected during photosynthetic cultivation, the authors assumed that similar reversions occurred in the cross-inactivated forms used to test heme *b*_L_–*b*_L_ electron transfer. Based on this assumption they raised concern that our kinetic traces of cross-inactivated _W_B–B^N^ [10] did not reveal heme *b*_L_–*b*_L_ electron transfer but rather originated from the pseudo-native contaminants. The results presented here do not support this view. The cells expressing _W_B_S_–B_N_ did not grow photosynthetically and the estimated frequency of reversion was very low. Consistent with this behavior, the kinetic traces of _W_B_S_–B_N_ showed no signs of native-like activity. Yet the kinetic traces of cross-inactivated ^N^B_S_–B_W_ matched the traces recorded earlier for the _W_B–B^N^. Clearly, the traces of all our cross-inactivated forms revealed electron transfer between the hemes *b*_L_. It follows that our original kinetic experiments with _W_B–B^N^ that were prepared using the *caps–caps* system [10] were free of any pseudo-native contaminants despite the fact that recombination frequency was larger in this system comparing to the *sphaer–caps* system.

It should be emphasized that the effect of selection of cells that expressed unfused cytochrome *b* in *R. sphaeroides* under photosynthetic conditions described in ref. [33] is consistent with our observations made for the *caps–caps* system [14]. However, we do not share a view that the photosynthetic selection in these cases reflects in vivo enforcement for monomeric electron transfer, as suggested [33]. While various reasons can be envisaged for this selection (see discussion in ref. [14]), it is clear that it took place irrespective of whether the original mutations within the fusion gene enforced the inter-monomer electron transfer within B–B or not.

It should also be emphasized that the reported by Hong et al. difficulties with interpretation of kinetic experiments [33] originate from the fact that they used photosynthetic growth conditions selecting recombined proteins to prepare the samples for kinetic analysis. We, on the other hand, specifically avoided this type of selection in our preparations.

The successful expression of hybrid cytochrome b_S_b and its assembly with other core subunits to form B_S_–B adds to B–B as another remarkable example of the overall structural plasticity of cytochrome *bc*_1_ design and a flexibility within the whole protein expression and assembly system (see [34] for discussion of this issue). However, like in the case of B–B and its derivatives, some distortions from the native structure cannot be ruled out. In this context, one may wonder how much these proteins, which all should undoubtedly be treated as model proteins, resemble native cytochrome *bc*_1_. The biochemical, spectroscopic and kinetic properties of these proteins (in particular the occurrence of all of the characteristic kinetic phases of electron transfer) allow us to be confident that the overall mode of operation follows the catalytic cycle of native cytochrome *bc*_1_. The fact that heme *b*_L_–*b*_L_ electron transfer was confirmed independently by the asymmetric forms of the cytochrome *bc*_1_-like fusion complexes coming from two different genetic systems (*caps–caps* and *sphaer–caps*) provides strong evidence in support of the notion that the heme–*b*_L_–*b*_L_ bridge forms an electronic connection available for inter-monomer electron transfer in cytochrome *bc*_1_.

The experimental evidence for the electron transfer between hemes *b*_L_ emerging from our studies and supported by other independent investigations [12] opens doors to discussions about the physiological significance of the intermonomer electron transfer for cytochrome *bc*_1_ operating in living cells. In this respect, one of the crucial aspects that needs detailed investigation concerns the ratio of intra-monomer vs inter-monomer electron transfer [1,9,35]. This ratio is likely to change in response to the changes in redox conditions and/or changes in the membrane potential. In addition, the ratio might be affected by certain mutations that inactivate or impair parts of the protein and are associated with the process of accumulation of mitochondrial mutations occurring in mitochondrially-coded cytochrome *b* subunit. Future studies with asymmetrically mutated forms of cytochrome *bc*_1_ should provide information on those and other related issues to advance our general understanding on the operation of this complex enzyme.

## Figures and Tables

**Table 1:** Assembly of B–B and B_S_–B complexes in *R. capsulatus* cells.

Name of fusion protein (*caps–caps* system)	Assembly of B–B	Name of fusion protein (*sphaer–caps* system)	Assembly of B_S_–B
B–B	+	B_S_–B	+
_W_B–B_W_	−	_W_B_S_–B_W_	+
^N^B–B^N^	−	^N^B_S_–B^N^	−
_W_B–B_N_	−	_W_B_S_–B_N_	+
^N^B–B_W_	−	^N^B_S_–B_W_	+
_W_B–B^N^	+	_W_B_S_–B^N^	+

W, N (subscript), and N (superscript) indicate position of mutation corresponding to G158W, H198N and H212N in cytochrome *b* subunit, respectively. “**+**” indicates assembly of the complex containing the fusion protein. “−” indicates lack of the fusion protein (for ^N^B_S_–B^N^, _W_B–B_W,_^N^B–B^N^, _W_B–B_N_) or the presence of dominant fraction of the complex with unfused protein (for ^N^B–B_W_) see ref. [14].
