# Supplementary material for: Catalytically-relevant electron transfer between two hemes bL in the hybrid cytochrome bc1-like complex containing a fusion of Rhodobacter sphaeroides and capsulatus cytochromes b
Source: Biochim Biophys Acta. 2013 Jun;1827(6):751–60. doi: 10.1016/j.bbabio.2013.02.007 (PMC4330944; doi:10.1016/j.bbabio.2013.02.007)
Supplement: Supplementary file 1 — Supplementary material. [file mmc1.pdf]

Supplementary data to:

Catalytically-relevant electron transfer between two hemes b_L in the hybrid cytochrome bc_1-like complex containing a fusion of *Rhodobacter sphaeroides* and *capsulatus* cytochromes *b*.

Monika Czapla, Ewelina Cieluch, Arkadiusz Borek, Marcin Sarewicz, Artur Osyczka

Construction of plasmid pMTS1-BS for the B_S complex expression.

The B_S complex was expressed from plasmid pMTS1-BS, a derivative of pMTS1 [1] in which *pet*B gene (encoding cytochrome *b* subunit) in *pet*ABC operon (coding for *Rhodobacter capsulatus* cytochrome bc_1 subunits) was exchanged on *fbc*B gene encoding cytochrome *b* subunit of *Rhodobacter sphaeroides* cytochrome bc_1. The all steps of pMTS1-BS construction are summarized in Fig. S1. The two plasmids: pPET1-BL containing *pet*ABC operon encoding all three subunit for cytochrome bc_1 of *Rb. capsulatus* and pBC9 [2] containing operon encoding subunits for cytochrome bc_1 of *Rb. sphaeroides* were used as a template for plasmid pMTS1-BS construction. First, using a methodology of PCR-based QuickChange Site-directed Mutagenesis (Stratagene), the restriction site for *Spe*I endonuclease was introduced into the noncoding DNA region between *pet*A and *pet*B genes of pPET1-BL, creating pPET1-BLS plasmid (Fig. S1A). The mutagenic oligonucleotides used in PCR reaction were CSpeI-F and CSpeI-R (Table 1S). In the next step the gen *fbc*B *of* pBC9 plasmid (contains operon for cytochrome bc_1 of *Rb. sphaeroides*) was PCR-amplified using primers SSpeI-F and SNotI-R (Table 1S), then inserted into the plasmid pCR-Blunt II TOPO (Invitrogen). This created plasmid TOPO-fbcB (Fig. S1B). Next the *Spe*I/*Not*I fragment of pPET1-BLS was exchanged with the *Spe*I/*Not*I DNA fragment from TOPO-fbcB creating pPET1-BS-Not plasmid (Fig. S1C). In the following step, using PCR and primers Stop-F and Stop-R (Table S1), the Stop codon was introduced into the end of *fbc*B gene of pPET1-BS-Not plasmid in the place of *Not*I restriction site, creating the pPET1-BS plasmid (Fig. S1D). In the last step *BstX*I/*Sfu*I DNA fragment of pMTS1 was exchanged with its counterpart from pPET1-BS which resulted in pMTS1-BS plasmid (Fig. S1E).

Construction of plasmid pMTS1-BSBST for the B_S-B complex expression.

The B_S-B complex was expressed from plasmid pMTS1-BSBST, a derivative of pMTS1 [1] in which *pet*B gene was replaced by a fusion gene *fbc*B/*pet*B (a hybrid of genes coding for *Rb. sphaeroides* and *Rb. capsulatus* cytochrome *b*). A summary of pMTS1-BSBST construction is displayed in Fig S2. The plasmids pPET1-BS-Not (Fig. S1C) and pUC-BLST [3] were used to create pMTS1-BSBST. First *Not*I/*Sfu*I DNA fragment of pUC-BLST was cloned between *Not*I/*Sfu*I restriction sites of pPET1-BS-Not creating pPET1-BSBST plasmid (Fig. S2A). Then the *BstX*I/*Sfu*I DNA fragment of pMTS1 was exchanged with its counterpart from pPET1-BSBST creating pMTS1-BSBST plasmid (Fig. S2B).

Introducing point mutations into the pMTS1-BSBST plasmid.

The series of plasmids pMTS1-BSBST containing various combinations of point mutations G158W, H198N, and H212N were generated in the following way. First desired mutations were introduced into pPET1-BS-Not and/or pUC-BLST (Fig. S2A). The mutations G158W and H212N were introduced into pPET1-BS-Not using a methodology of PCR-based QuickChange Site-directed Mutagenesis (Stratagene) and the mutagenic oligonucleotides: G158W-F, G158W-R and H212N-F, H212N-R (Table S1). To create pUC-BLST containing H212N or H198N, the appropriate DNA fragments from the pPET1 templates carrying those mutations were transferred using appropriate restrictions cuts and ligations. The point mutation G158W was introduced directly into pUC-BLST using PCR-based mutagenesis [3]. The strategy of combining the mutagenized fragments of pPET1-BS-Not and pUC-BLST to construct expression plasmids pMTS1-BSBST containing various combinations of mutations follows the general approach described in Fig. S2.

DNA sequence and correct size of all constructs were verified at the stage of their preparation. In addition, DNA sequence and correct size of all expression vectors were verified after the re-isolation of plasmids from *Rb. capsulatus* cells.

References

[1] E. Atta-Asafo-Adjei, F. Daldal, Size of the amino acid side chain at position 158 of cytochrome *b* is critical for an active cytochrome *bc*$_1$ complex and for photosynthetic growth of *Rhodobacter capsulatus*, Proc. Natl. Acad. Sci. USA 88 (1991) 492-496.

[2] C.-H. Yun, R. Beci, A.R. Crofts, S. Kaplan, R.B. Gennis, Cloning and DNA sequencing of the *fbc* operon encoding the cytochrome *bc*$_1$ complex from *Rhodobacter sphaeroides*. Characterization of *fbc* deletion mutants and complementation by a site-specific mutational variant, Eur. J. Biochem. 194 (1990) 399-411.

[3] M. Czapla, A. Borek, M. Sarewicz, A. Osyczka, Fusing two cytochromes *b* of *Rhodobacter capsulatus* cytochrome *bc*$_1$ using various linkers defines a set of protein templates for asymmetric mutagenesis, Protein Eng. Des. Sel. 25 (2012) 15-25.

Fig. S1

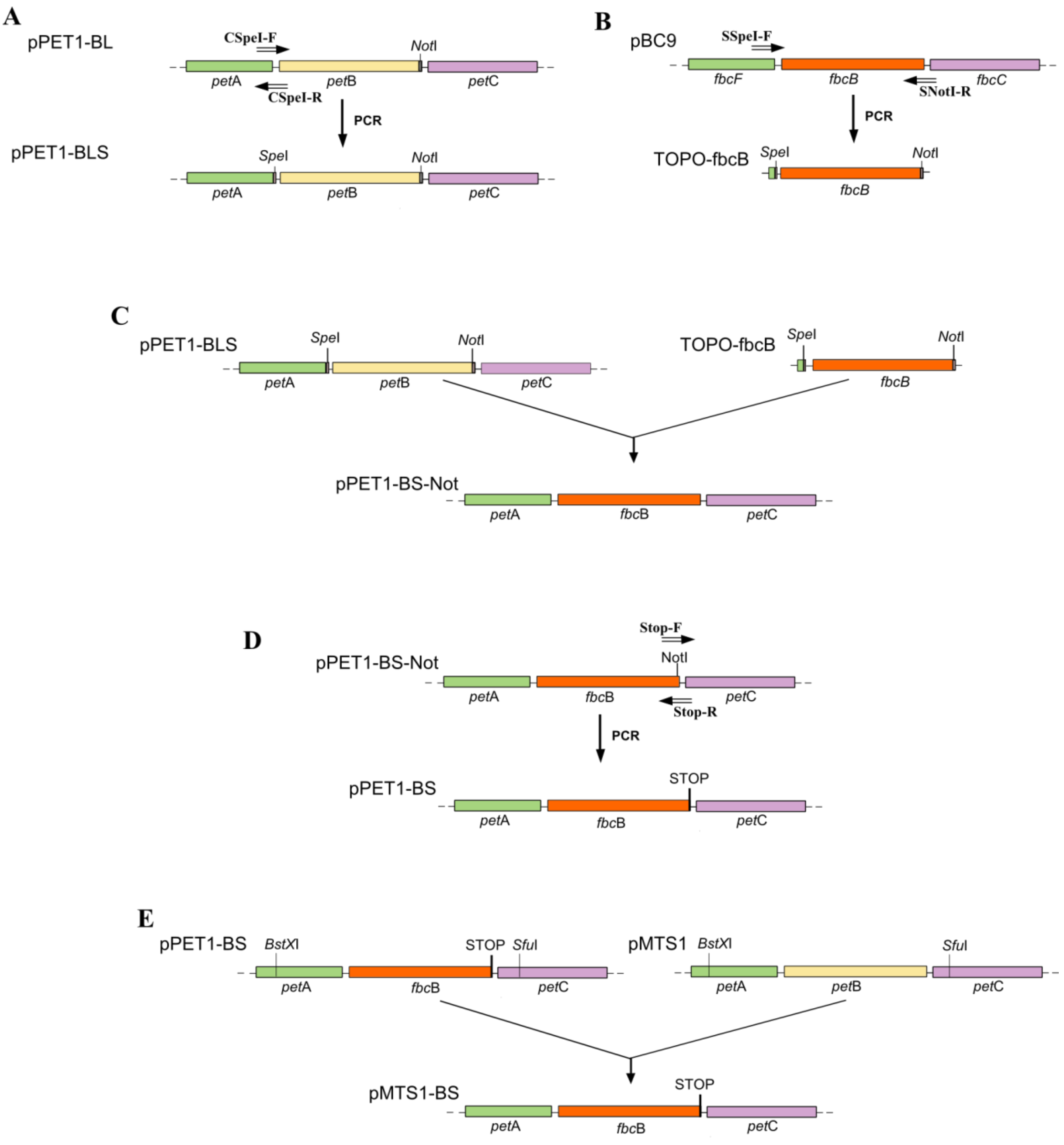

Fig. S1 Summary of construction the expression plasmid pMTS1-BS. Horizontal arrows indicate position of mutagenic primers. Vertical lines indicate restriction sites used for plasmid construction.

Fig. S2

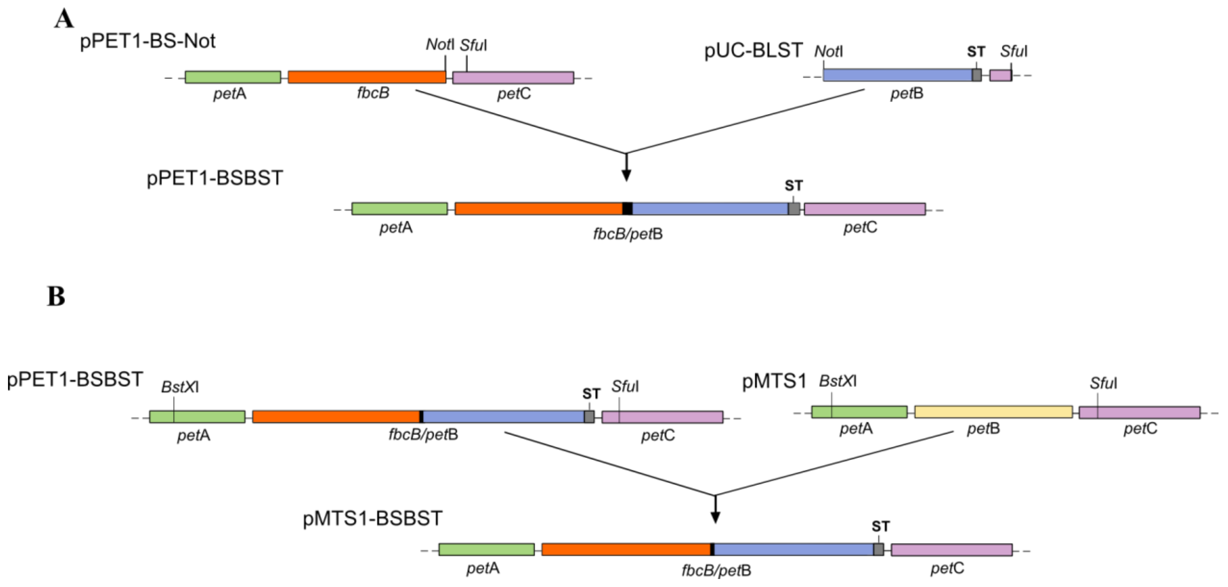

Fig. S2 Summary of construction the expression plasmid pMTS1-BSBST. Vertical lines indicate restriction sites used for plasmid construction. Black box denotes the linker (the sequence of the linker is shown in Fig. 3). Grey box denotes the sequence coding for the strep-tag (ST).

Fig. S3

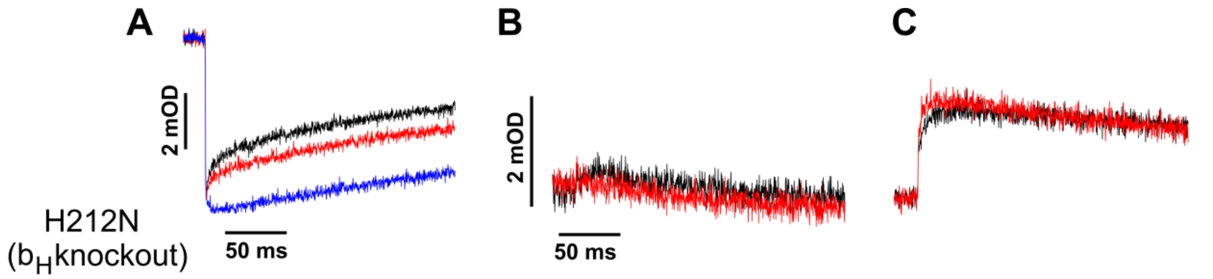

Fig. S3. Light-induced electron transfer in chromatophore membranes containing mutant H212N (b_H knockout). Kinetic transients were recorded at 550-540 nm for cytochrome *c* (**A**), 560-570 nm for heme b_H (**B**), and 566 – 573 nm for heme b_L (**C**) at pH=7 and ambient potential of 100 mV. Color code: no inhibitor, black; antimycin, red; myxothiazol, blue. The horizontal and vertical scales in C are as in B.

Table S1. Primers used in PCR reactions

Name of mutagenic primer	5’ → 3’ DNA sequence of primer
CSpeI-F	CAAGCTGGGCTGACTAGTGGGGAAAGACAC
CSpeI-*R*	GTCTTTCCCCACTAGTCAGCCCAGCTTGATCG.
SSpeI-F	CCAGCTCGGGTAACTAGTGGAACACGCCATGTCC
SNotI-R	CGTCACTGTCCTTTGCGGCCGCCTGCCACGACCG
Stop-F	CGGTCGTGGCAGAGTAAGGAAAGGAACCGAC
Stop-R	CGGTTCCTTTCCTTACTCTGCCACGACCG
G158W-F	GATGTCGTTCTGGTGGGCCACCGTGATCACC
G158W-R	GATCACGGTGGCCCACCAGAACGACATCTGG
H212N-F	CTCGTGGCCATCAACATCTGGGCCTTCC
H212N-R	GAAGGCCCAGATGTTGATGGCCACGAGGGC
